# Remnant cholesterol and atherosclerotic cardiovascular disease: Metabolism, mechanism, evidence, and treatment

**DOI:** 10.3389/fcvm.2022.913869

**Published:** 2022-10-17

**Authors:** Kexin Wang, Rui Wang, Jiaxin Yang, Xiaoli Liu, Hua Shen, Yan Sun, Yujie Zhou, Zhe Fang, Hailong Ge

**Affiliations:** ^1^Department of Cardiology, Beijing Anzhen Hospital, Capital Medical University, Beijing, China; ^2^Jiangxi Provincial People’s Hospital, The First Affiliated Hospital of Nanchang Medical College, Nanchang, China; ^3^Department of Cardiology, Beijing Daxing Hospital, Capital Medical University, Beijing, China

**Keywords:** remnant cholesterol, lipoprotein, atherosclerosis, cardiovascular disease, general population

## Abstract

This review aimed to summarize the evidence of elevated remnant cholesterol and the risks of atherosclerotic cardiovascular disease (ASCVD) and to search for further guidance in clinical therapy. The lipids-lowering treatments such as statins and ezetimibe targeted on low-density lipoprotein cholesterol (LDL-C) have always been the first-line therapy for ASCVD. However, even after statins or new lipid-lowering drugs lowered LDL-C to recommended concentrations, and with other risk factors well-controlled, such as high blood pressure, the risks of developing ASCVD remained. Remnant cholesterol (RC) referred to the cholesterol contained in all remnant lipoprotein particles, which was the cholesterol in the hydrolyzed very-low-density lipoprotein and intermediate-density lipoprotein in the fasting state, and the cholesterol in the chylomicron remnants in the postprandial state. Evidence from *in vitro* and animal pathogenic mechanisms studies, epidemiology, and genetic studies all indicated that RC played an important role in predicting the incidence of ASCVD. As a new indicator to reflect atherosclerosis, especially when LDL-C has been controlled to a recommended level, RC was considered as a priority treatment target for people at high risk of ASCVD. The use of statins, fibrates, APOC3 inhibitors, PCSK9 inhibitors, and omega-3 fatty acids to reduce RC levels in the plasma may provide long-term benefits. However, the standardized detection of RC was still controversial, and more studies on appropriate treatments of elevated RC are urgently needed. These positive trials may benefit more patients at high ASCVD risks worldwide in the future.

## Introduction

Over the past years, atherosclerotic cardiovascular disease (ASCVD) has become a chronicle epidemic disease. Despite significant progress in the prevention and treatment of ASCVD, it remains the leading cause of death worldwide. The report on the global burden of ASCVD from 1990 to 2019 showed that in 2019, nearly 197 million people suffered from ASCVD, and 9.14 million people died from ischemic heart disease (IHD) (1). Dyslipidemia has always been a major causative factor in the incidence and progress of ASCVD. Moreover, high systolic blood pressure (54.6%), high low-density lipoprotein cholesterol (LDL-C) level (46.6%), smoking (23.9%), high blood glucose (23.8%), and obesity/overweight (22.7%) were the global top five leading influencing factors of the ASCVD disability-adjusted life year (DALY) (2). Thus, the lipids-lowering treatments such as statins and ezetimibe targeted on LDL-C have always been the first-line therapy for ASCVD. In recent years, compared with the 2016 European Society of Cardiology (ESC) guideline, the new guideline based on more research evidence has been greatly updated. For the blood lipids management in patients with ASCVD, more strict target recommendations should be given, such as LDL-C should be reduced to <1.4 mmol/L (55 mg/dl), and more than 50% compared with baseline (3, 4).

However, even after statins or new lipid-lowering drugs lowered LDL-C to recommended concentrations, and with other risk factors well-controlled, such as high blood pressure, the risks of developing ASCVD remained (5, 6). When LDL-C was less than 2.6 mmol/L, atherogenic dyslipidemia was characterized by: high levels of triglyceride (TG) (TG ≥1.70 mmol/L) and low levels of high-density lipoprotein cholesterol (HDL-C) (women <1.29 mmol/L, men <1.03 mmol/L), which were common in diabetes, obesity, metabolic syndrome, and ASCVD patients with renal failure disease (7). Elevated TG levels in plasma indicated an increase in chylomicron (CM) or very-low-density lipoprotein (VLDL) particles, also known as triglyceride-rich lipoproteins (TRLs) (8). The metabolized TRLs were also called triglyceride-rich remnant lipoprotein particles (RLPs), which were listed as an emerging atherogenic risk factor by the American Heart Association (AHA) in 2001 (9). There was evidence showing that it was the cholesterol components of RLPs, the remnant cholesterol (RC), rather than TG, that increased ASCVD risks (10). In this review, we defined RC as the cholesterol contents of a subset of the TRLs called remnants, i.e., CM remnants in the non-fasting state, and VLDL and intermediate-density lipoprotein (IDL) in the fasting state (11). The review aimed to summarize the evidence of elevated RC and the risks of ASCVD, and to search for further guidance in clinical therapy.

## Metabolism and measurements of remnant cholesterol

**FIGURE 1:**
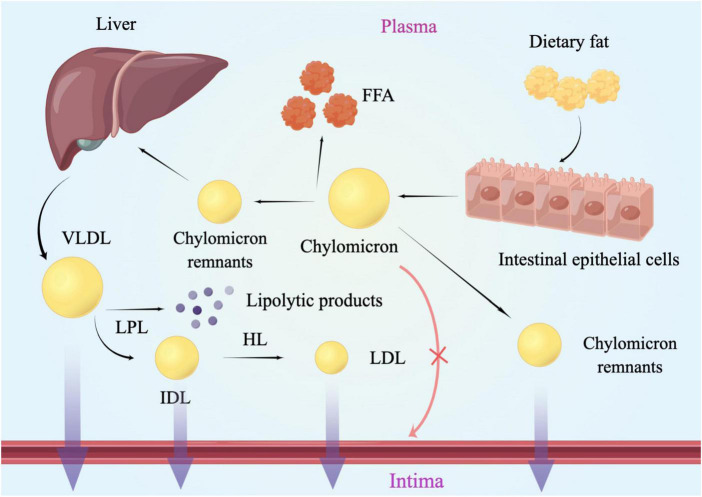
Lipoproteins metabolism and the arterial wall. Chylomicrons of different sizes needed to be converted into metabolic remnants, while VLDL needed to be metabolized to LDL to be cleaned. After being digested, CM and VLDL entered into the plasma and began to be lipolysis by lipoprotein lipase. Then CM remnants and VLDL remnant-like particles with higher density and smaller molecular weight were formed, respectively. Lipoproteins larger than 75 nm were not easily available to get across the arterial intima, such as chylomicrons. LPL, lipoprotein lipase; HL, hepatic lipase; VLDL, very-low-density lipoprotein; IDL, intermediate-density lipoprotein; LDL, low-density lipoprotein; FFA, free fatty acid; RC, remnant cholesterol.

The hydrophobic properties of triglycerides as well as its lipolysis products underlie the association between TG and atherosclerosis. Inefficient triglyceride transport has led to the accumulation of plasma TRLs and their partial lipolysis products, also known as remnants (12). RC referred to the cholesterol contained in RLPs particles, which was the cholesterol in the VLDL and IDL in the fasting state, and the cholesterol in the CM remnants in the postprandial state (13). Chylomicrons of different sizes needed to be converted into metabolic remnants, while VLDL needed to be metabolized to LDL to be cleaned (14). After being digested, CM and VLDL entered into the plasma and began to be lipolysis by lipoprotein lipase (LPL), which needed to be activated under the activation of apolipoprotein C II. As the research showed before, apoC-II and apoA-5 could promote the activity of LPL (15, 16), whereas apoC-I, apoC-III and apoE could inhibit LPL activity (17). The relative content of apoE and apoC-III could be the key determinant of granules undergoing receptor-mediated catabolism. Residual clearance was delayed in patients with apoE dysfunction [such as type III hyperlipidemia (18)], while apoC-III has been confirmed as an inhibitor of TRL clearance (19). After gradually losing TG, phospholipid, apolipoprotein A (APOA), and apolipoprotein C (APOC), CM remnants and VLDL remnant-like particles with higher density and smaller molecular weight were formed, respectively (20). As a product of VLDL hydrolysis, IDL was also a kind of RC (21) (Figure 1).

The measurements of RC in the plasma were generally divided into two major types: the calculated methods and the directly measured methods (22, 23). The calculated RC (CRC) was defined as total cholesterol (TC) minus LDL-C minus HDL-C (24), which did not require special detection equipment, and was more convenient and easier to implement in clinical diagnosis and therapy. However, it was worth noting that for the detection of LDL-C in the formula, when TG < 5.6 mmol/L, the Friedewald equation was used, while when TG ≥ 5.6 mmol/L, the method of direct detection was used (25, 26). In addition to CRC, the directly measured RC (MRC) had a variety of detection methods, including direct automatic assay, immune separation, and ultracentrifugation. The direct automated assay used enzymes and surfactants to detect cholesterol content in CM and VLDL (27, 28). The immune separation method was detecting the cholesterol content of a small subunit on TRLs, and the cholesterol content of CM remnants and VLDL remnants were measured after the removal of ApoA1 and ApoB100 by antibodies. Chylomicron remnants can be quantitatively evaluated by serum apolipoprotein B-48 concentration (8). The directly measured methods were complicated and the price was relatively expensive, which limits the large-scale clinical use (29). Jepsen et al. and Cao et al. have compared the CRC and MRC in cardiovascular outcomes of patients with IHD. The results showed that both RC were considered to be associated with an increased risk of major adverse cardiovascular events (MACEs) (22, 30), and the calculated RC showed a stronger correlation (30).

## Epidemiologic studies about remnant cholesterol levels and atherosclerotic cardiovascular disease risks

### Non-fasting remnant cholesterol and atherosclerotic cardiovascular disease

Since the fasting state means no diets at least for 8 h, which is usually implied in the early morning, abundant research investigated the relationship between non-fasting lipids levels and ASCVD. So far, many prospective studies and clinical trials have demonstrated that elevated non-fasting RC levels can predict the increased risk of the incidence and development of arteriosclerotic cardiovascular disease, which is more capable than LDL-C levels (10, 13, 22, 31, 32). A prospective study of 73,513 patients from the Copenhagen General Population Study (the CGPS) and the Copenhagen Ischemic Heart Disease Study (the CIHDS) with 34 years of follow-up found that every 1 mmol/L increase in non-fasting RC was associated with a 40% increase in the risk of ASCVD. However, for the levels of LDL-C, every 1 mmol/L increase in RC was only associated with a 10% increase in the risk of ASCVD (31). It has confirmed a higher risk of non-fasting RC than LDL-C. Another survey of 82,890 people from the Copenhagen Heart Center showed that with an increased RC level, patients had an increased risk of all-cause mortality, which was not associated with an increased LDL-C level. And compared with the patients with RC levels less than 0.5 mmol/L (19 mg/dl), the risk of all-cause mortality in patients with RC more than 5 mmol/L (58 mg/dl) was 1.4 times higher (27). Furthermore, a recent prospective study involving 2,973 patients with acute myocardial infarction or ischemic stroke found that patients with RC < 0.8 mmol/L could reduce the incidence of recurrent cardiovascular events by at least 20% (10).

### Fasting remnant cholesterol and atherosclerotic cardiovascular disease

**FIGURE 2:**
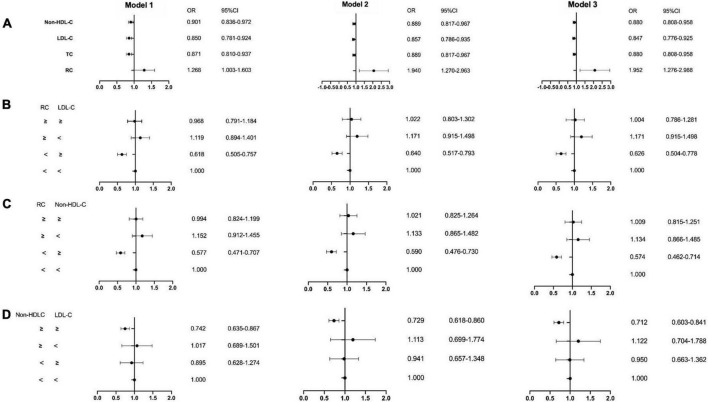
The result of multivariable logistic regression analyze. Model 1: age, gender, hypertension, hypercholesterolemia, smoking, diabetes, BMI; Model 2: Model 1, SBP, DBP, FBG, HbA1C, HDL-C, TG; Model 3: Model 2, creatinine, uric acid, hs-CRP, BNP, WBC, homocysteine. The medians of the RC, LDL-C and non-HDL-C index were calculated to divide all patients into two different groups: low (less than the medians) and high (equal to or greater than the medians). Then patients were categorized into four groups according to having a low or high RC index and LDL-C, RC and non-HDL-C, non-HDL-C and LDL-C as follows: low/low, low/high, high/low, and high/high. **(A)** Non-HDL-C, LDL-C, TC and RC; **(B)** RC and LDL-C group; **(C)** RC and non-HDL-C group; **(D)** non-HDL-C and LDL-C group. LDL-C, low-density lipoprotein cholesterol; HDL-C, high-density lipoprotein cholesterol; RC, remnant cholesterol; OR, odds ratios; CI, confidence intervals. Modified from Wang et al. (34).

Castañer et al. investigated the relationship between lipid levels in plasma and MACEs (e.g. myocardial infarction, stroke, or cardiovascular death) in the PREDIMED study, a cohort having the high-risk cardiovascular disease, which included 6,901 individuals. They found that every 0.26 mmol/L increase in RC leads to a 21% increase in the risk of cardiovascular events. According to the multivariate adjustment analysis in this research, RC but not LDL-C level, was associated with cardiovascular events in overweight or obese subjects, independent of lifestyle and other risk factors. And RC at 0.78 mmol/L was defined as the cut-off of higher risks of ASCVD (11). Elshazly et al. analyzed the data from 5,754 patients with ASCVD who underwent serial intravascular ultrasonography and compared the development of atheroma volume and 2 years follow-up MACEs at different RC levels. It has been demonstrated that in statin-treated ASCVD patients, the elevated RC was positively correlated with coronary atherosclerosis volume progression (+0.53 ± 0.26 vs. −0.15 ± 0.25%, *p* < 0.001) and the MACEs (23 vs. 14%, log-rank *p* < 0.001), which was regardless of conventional lipid parameters, C-reactive protein, or other clinical risk factors (33). Furthermore, we previously conducted a retrospective study including 12,563 patients, in which RC was calculated as TC minus LDL-C minus HDL-C (34). The study reported that the elevated fasting RC level in the coronary artery disease (CAD) group was statistically significantly different from the non-CAD group (0.51 vs. 0.49, *p* < 0.001), and further analysis found that RC was an independent risk factor for ASCVD, with a causal odds ratio of 1.952 (CI = 1.276–2.988, *p* = 0.002). In addition, according to the concordance/discordance groups analysis, the level of RC seemed more capable to predict the ASCVD risks than the LDL-C level (Figure 2), which reached the same conclusion as Castañer et al. (11).

So far, epidemiological research has confirmed that triglycerides is an independent risk factor for atherosclerotic coronary heart disease (35). The relationship between triglycerides and coronary heart disease was statistically significant when the adjusted multivariate logistic regression models were performed on triglycerides and other factors (14, 36). And the level of RC seemed more clinically predictive than TG (37). As the research mentioned above showed, both non-fasting and fasting RC could lead to a higher risk in the incidence and progress of ASCVD. Different from the fasting RC, the non-fasting RC also contains the cholesterol in chylomicron remnants. More mechanisms will be discussed later.

## Mechanisms

**FIGURE 3:**
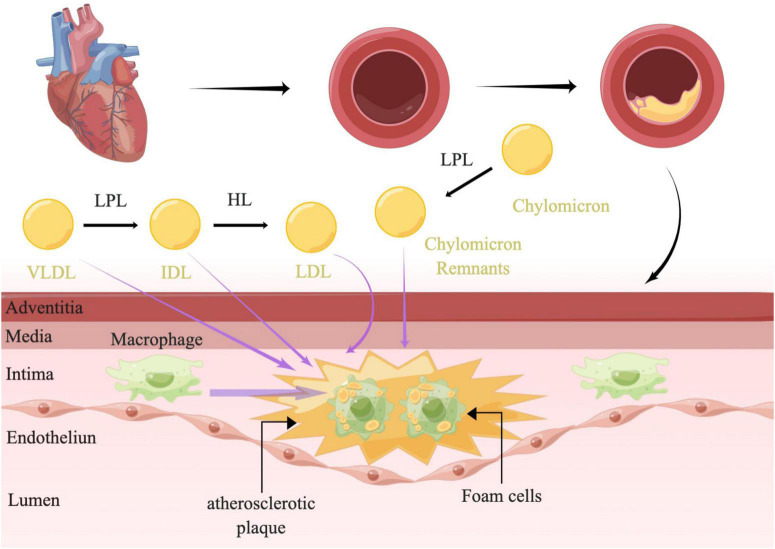
The mechanism of atherosclerosis. LPL along the capillary lumen surface hydrolyzed VLDL, resulting in VLDL remnants/IDL particles and lipolysis products. The IDL particles were further catabolized into LDL by HL. LPL could also hydrolyzed chylomicrons to produce chylomicron remnants. TRLs and their remnants readily penetrate the arterial wall and can be taken up by scavenger receptors on macrophages directly without oxidative modification, leading to formation of foam cells and atherosclerotic plaque development. TRLs, triglyceride-rich lipoproteins; LPL, lipoprotein lipase; HL, hepatic lipase; VLDL, very-low-density lipoprotein; IDL, intermediate-density lipoprotein; LDL, low-density lipoprotein; RC, remnant cholesterol.

The plasma levels of RC were shown to be inversely correlated with HDL-C (38), which could prevent inflammation and oxidative stress and promote cholesterol efflux to reduce lesion formation (39). Although the association was attenuated after correction for the level of HDL-C, a consensus was emerging based on abundant clinical and population data and genetic studies that fasting and non-fasting plasma triglycerides were predictive of ASCVD (10, 38). As studies have reported before, damage to the vascular endothelial cells could promote the development of atherosclerosis. Nordestgaard et al. demonstrated that the most necessary condition for TRLs promoting atherosclerosis was the ability to invade the arterial intima, which was directly determined by the size of lipoproteins. Lipoproteins larger than 75 nm were not easily available to get across the arterial intima, such as chylomicrons (40) (Figure 1). While lipoproteins of 20–60 nm could easily get across the arterial intima (41). A human and animal study has also shown that medium-sized TRLs could enter into the intima of the arterial wall, however, much slower than the LDL particles (42). TRLs could be phagocytosed by peripheral blood macrophages through the VLDL receptor, APOB 48 receptor, or LDL receptor-related protein (43) without modification (44). Moreover, saturated fatty acids (FAs) and phospholipids containing oxidized FAs that were generated by lipolysis of TRLs could be taken up into parenchymal cells and could induce maladaptive inflammatory responses (12). As the remnants were digested, the triglycerides, proteins, and phospholipids were degraded, while a large number of undigested cholesterol droplets remained in macrophages (41). Indeed, it was the undigested cholesterol droplets, not triglycerides that could accumulate in intimal foam cells (42, 45). Elevated RC in the plasma could promote the lipids invasion of the arterial wall. After passing through the vascular endothelial layer, the RC could accumulate and be absorbed by macrophages and smooth muscle cells (SMC), further became the foam cells, and finally transformed to the part of atherosclerotic plaques (8, 46–48) (Figure 3). In addition, it has a stronger atherosclerotic ability due to the needless oxidative modification. Once RC entered the intima, it could attach to the extracellular proteoglycans, preferentially to LDL-C (45). Meanwhile, RC could increase the production of reactive oxygen species and trigger endothelial cell dysfunction (49). Lipoprotein lipase released free fatty acids (FFAs) and monoacylglycerols during the hydrolysis of RC, which may lead to local inflammation and affected the formation and progression of atherosclerosis (50). Furthermore, RC could also promote platelet aggregation and microthrombus formation by accelerating the formation of prothrombinase complex and upregulating the expression of plasminogen activator inhibitor-1 and its antigens (51, 52).

A study showed that the level of RC in patients in the non-fasting state was 0.2 mmol/L higher than that in the fasting state (53). The reason could be that TG was hydrolyzed into FFAs and diglycerides in the stomach and proximal small intestine. Free fatty acids could be taken up by muscle cells and adipocytes, while cholesterol-rich chylomicrons remnants needed to be cleared by binding to LDL receptor or LDL receptor-associated protein-1 through liver APOE (30). Most studies detect RC in the non-fasting state, but the fasting state is also extremely important for accurately judging inherited abnormal lipoprotein metabolism.

## Genetic studies

**FIGURE 4:**
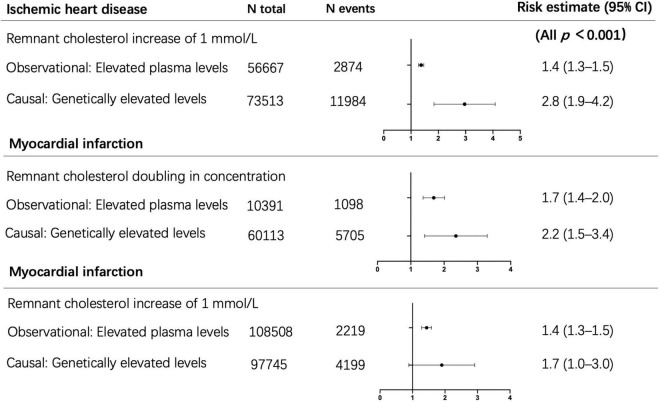
Observational and causal (by use of genetics) associations of elevated remnant cholesterol with risk of ischemic heart disease and myocardial infarction. Top section adapted from Varbo et al. (10). Middle section adapted from Jørgensen et al. (58). Bottom section adapted from Nordestgaard et al. (59). N, number.

Plasma levels of lipids, such as triglycerides or RC, were determined by common or rare genetic variants (54, 55), and lifestyle factors such as diet, obesity, alcohol consumption, and physical activity (56). It suggested that lifetime exposure to elevated RC levels caused by genetic abnormalities was associated with a greater risk of cardiovascular disease. In the epidemiological etiology inference, the method of Mendelian randomization has always been applied to exclude confounding factors between lipids and ASCVD. Therefore, genetic evidence could provide insight into whether lifelong raised TRLs and RC are causally connected with low-grade inflammation, ASCVD, and more MACEs. Tada et al. illustrated that RC was associated with CAD in the general population, and serum RC levels were associated with the incident and progress of ASCVD in patients with familial hypercholesterolemia (FH) (57). Varbo et al. screened 15 kinds of genes that may affect blood lipid levels and genotyped 73,413 cases in the Copenhagen research to observe the incidence of ASCVD of each genotype. The results showed that each 1 mmol/L increase in the non-fasting RC was associated with a 2.8-fold increase in the risk of ASCVD, which suggested that the non-fasting RC was a risk factor of ASCVD, independently of LDL-C (10) (Figure 4, top section). In addition, the elevated RC was associated with a 2.2-fold increased risk of myocardial infarction when there was a variant in the APOA5 (apolipoprotein A5) gene, and the corresponding observational estimate was 1.7 times (58)(Figure 4, middle section). Meanwhile, Nordestgaard et al. analyzed four gene mutations that specifically affected the RC levels, including APOA5, GCKR, LPL, and TRIB1, and compared the causal odds ratio and observed hazard ratio of patients exposed to each different gene mutation. The results found that every 1 mmol/L increase in RC, the causal odds ratio for myocardial infarction increased 1.7 times and the observed hazard ratio increased 1.4 times (59) (Figure 4, bottom section). Finally, high concentrations of RC were genetically associated with increased low-grade inflammation, which was not the same as the high concentrations of LDL-C. Thus, it was suggested that an inflammatory component of atherosclerosis was driven by TRLs (24).

## New treatments of elevated remnant cholesterol

As the 2021 ESC guideline recommends (3), lifestyle interventions are fundamental to control elevated TRLs levels, including weight loss, increased physical activity, limiting alcohol intake, and avoiding fructose or high-carbohydrate foods. And for the drug interventions that lowered RC level were typically designed to lower plasma triglycerides. For the patients with hypertriglyceridemia (TG > 5.6 mmol/L or >500 mg/dl), if the TG level is more than 2.3 mmol/L (or >200 mg/dl), high-intensity statins are recommended. In patients with type 2 diabetes with or without ASCVD, if the TG level is more than 2.3 mmol/L (or >200 mg/dl) after statin treatments, statins or ezetimibe with fenofibrate could be considered, which may receive more macrovascular and microvascular benefits. Although the guideline has not directly recommended decreasing the RC level in the plasma, the awareness of controlling other blood lipid components except for LDL-C in ASCVD patients is arousing, which will undoubtedly benefit people who meet LDL-C targets but still have residual cardiovascular risks. For the new treatments of elevated RC, there were abundant new studies being conducted, which mainly concentrated on statins, fibrates, and omega-3 fatty acids (OM_3_FAs). The PREVAIL US Trial showed that all statins could effectively reduce the level of RC in ASCVD patients, and pitavastatin was stronger than other statins (60). For the fibrates, Tsunoda et al. demonstrated that fibrates could also reduce the RC levels. Compared with placebo, it could not only reduce TG, but also RC levels in patients with type 2 diabetes (61). And the ACCORD-Lipid study showed that the fenofibrate was effective for ASCVD risk reduction in patients with type 2 diabetes, which have already treated with statins (62). Ishibashi et al. also illustrated that as a novel selective peroxisome proliferator-activated receptor α modulator (SPPARMα) that possessed unique PPARα activity and selectivity, pemafibrate could lowering RC as well as TG (63). However, in the Helsinki Heart Study, gemfibrozil has reduced the risk of ASCVD by 32% without any significant effects on preventing stroke or all-cause mortality (64). Again, in the FIELD study (65), fenofibrate did not demonstrate a reduction in the rate of the coronary events (coronary heart disease death or non-fatal myocardial infarction). Furthermore, a meta-analysis of 10 trials enrolling 77,917 participants showed that low-dose OM_3_FAs supplementation was not associated with significant cardiovascular benefits (66). But the REDUCE-IT trial changed this view. In patients with a history of cardiovascular disease, diabetes, or other risk factors, after statin treatments, the TG level was 1.52–5.63 mmol/L, and the LDL-C level was 1.06–2.59 mmol/L. High-dose (4 g/day) icosapent ethyl was given after stains, and the risk of ASCVD was significantly reduced by 25% after 4.9 years of follow-up (67).

Insights from genetic studies have also given us new prospects in therapeutic drug targeting, and the results are worth looking forward to, such as angiopoietin-like protein 4 antibodies, APOC3 inhibitor, and APOB antisense therapy (59, 68–70). For example, antisense inhibition of APOC3, which encoded apolipoprotein C3, has indicated promising results regarding the reduction of triglyceride levels in preclinical models and a phase I clinical trial (68, 71). However, before any of these potential new therapies for lowering RC levels reach clinical application, they should be demonstrated to be effective without any essentially side effects, either as a supplement to statin therapy or used alone in statin-intolerant patients.

## Conclusion and future directions

In conclusion, dyslipidemia was considered to be the main risk factor of ASCVD. Evidence from *in vitro* and animal pathogenic mechanisms studies, epidemiology, and genetic studies all indicated that RC plays an important role in predicting the incidence of ASCVD. As a new indicator to reflect atherosclerosis, especially when LDL-C has been controlled to a recommended level, RC was considered as a priority treatment target for people at high risk of ASCVD. Furthermore, stains were demonstrated to be effective in reducing cardiovascular disease and MACEs with few essential side effects. Meanwhile, the use of APOC3 inhibitors, PCSK9 inhibitors, and omega-3 FAs to reduce RC levels in the plasma may provide long-term benefits. However, the standardized detection of RC was still controversial, and more studies on appropriate treatments of elevated RC are urgently needed. Most desirable was a large randomized placebo-controlled trial in patients with mild-moderately elevated TG with recommended controlled LDL-C, treated with a potent statin and another RC lowering drug. These positive trials may benefit more patients at high ASCVD risks worldwide in the future.

## Author contributions

KW carried out the experiments, acquired the data, and wrote the first draft of the manuscript. RW carried out the experiments and wrote sections of the manuscript. JY, XL, HS, and YZ recruited the subjects, performed the patients’ assessments, and critically reviewed the manuscript for intellectual content. KW, ZF, and YS performed the statistical analyses. HG conceived and designed the study and handled funding and supervision. All authors contributed to the article and approved the submitted version.

## Abbreviations

ASCVD: atherosclerotic cardiovascular disease
CAD: coronary artery disease
RC: remnant cholesterol
TC: total cholesterol
TG: triglyceride
LDL-C: low-density lipoprotein cholesterol
HDL-C: high-density lipoprotein cholesterol
FFA: free fatty acid
ACS: acute coronary syndrome
STEMI: ST-segment elevation myocardial infarction
NSTEMI: non-ST-segment elevation myocardial infarction
LPL: lipoprotein lipase
HL: hepatic lipase
Apo: apolipoprotein
APOA5: apolipoprotein A5
APOC3: apolipoprotein C3.
